# Reactive nitrogen species act as the enhancers of glutathione pool in embryonic axes of apple seeds subjected to accelerated ageing

**DOI:** 10.1007/s00425-024-04472-5

**Published:** 2024-07-12

**Authors:** Tyminski Marcin, Ciacka Katarzyna, Krasuska Urszula

**Affiliations:** Department of Plant Physiology, Institute of Biology, Warsaw University of Life Sciences-SGGW, Nowoursynowska 159, 02-776 Warsaw, Poland

**Keywords:** Nitric oxide derivatives, Glutathione peroxidase-like, Glutathione reductase, Reactive oxygen species, Redox potential

## Abstract

**Main conclusion:**

Reactive nitrogen species mitigate the deteriorative effect of accelerated seed ageing by affecting the glutathione concentration and activities of GR and GPX-like.

**Abstract:**

The treatment of apple (*Malus domestica* Borkh.) embryos isolated from accelerated aged seeds with nitric oxide-derived compounds increases their vigour and is linked to the alleviation of the negative effect of excessive oxidation processes. Reduced form of glutathione (GSH) is involved in the maintenance of redox potential. Glutathione peroxidase-like (GPX-like) uses GSH and converts it to oxidised form (GSSG), while glutathione reductase (GR) reduces GSSG into GSH. The aim of this work was to investigate the impact of the short-time NOx treatment of embryos isolated from apple seeds subjected to accelerated ageing on glutathione-related parameters. Apple seeds were subjected to accelerated ageing for 7, 14 or 21 days. Isolated embryos were shortly treated with NOx and cultured for 48 h. During ageing, in the axes of apple embryos, GSH and GSSG levels as well as half-cell reduction potential remained stable, while GR and GPX-like activities decreased. However, the positive effect of NOx in the vigour preservation of embryos isolated from prolonged aged seeds is linked to the increased total glutathione pool, and above all, higher GSH content. Moreover, NOx increased the level of transcripts encoding GPX-like and stimulated enzymatic activity. The obtained results indicate that high seed vigour related to the mode of action of NO and its derivatives is closely linked to the maintenance of higher GSH levels.

**Supplementary Information:**

The online version contains supplementary material available at 10.1007/s00425-024-04472-5.

## Introduction

Excess reactive oxygen species (ROS), in conjunction with the defective antioxidant system, are factors contributing to ageing (Kurek et al. 2019). Seed ageing is a time-dependent, progressive physiological process that generates various economic and ecological problems. Changing and unpredictable environmental conditions fuel the ever-growing interest in the issue of oxidative stress in plants. Thus, the topic linked to seed ageing should be constantly expanded with new aspects.

ROS and reactive nitrogen species (RNS), including nitric oxide (NO), are regulatory compounds implicating seed physiology (Bailly 2019; Ciacka et al. 2022b). Considering the complex chemistry and reactivity of molecules derived from the NO radical form (^•^NO), the term “RNS” is limited. Some reactive species formed from NO are compounds that do not possess nitrogen in their structure, e.g. CO_3_^•−^ (Hughes 2008; Möller et al. 2019). Therefore, for the purposes of this publication, according to the biochemistry of NO, we will consider RNS as NO and related species/compounds (Hughes 2008). In the presence of oxygen and/or superoxide anion (O_2_^•−^), NO forms derivatives, including peroxynitrite (ONOO^−^) and its protonated form — peroxynitrous acid (ONOOH) (Stamler et al. 1992 and citations therein). The hydrophobic environment accelerates the reaction of NO with oxygen, resulting in the formation of toxic nitrogen dioxide (NO_2_), which, may be further converted to dinitrogen trioxide (N_2_O_3_) in the presence of NO. Recently, the presence of nitroxyl (HNO), a signalling molecule, was confirmed in living cells, in the model plant Arabidopsis (*Arabidopsis thaliana* (L.) Heynh.) (Arasimowicz-Jelonek et al. 2022). Depending on the structure and the number of electrons in the molecule, some RNS act as oxidising, nitrosating, or nitrating agents, which may react with various molecules, such as amines and thiols (Hughes 2008; Möller et al. 2019). Thus, the physical properties of NO, such as its small size, high diffusion rate, and lipophilicity, together with electron-dependent reactivity, make NO a molecule significantly involved in metabolism, acting as a protector or oxidiser (Stamler et al. 1992; Hughes 2008).

The deteriorative impact of overaccumulated ROS on seed vigour is well described (Kurek et al. 2019). ROS are responsible for the irreversible oxidation of essential cellular components such as proteins and nucleic acids. This is of particular importance since both proteins and RNA are accumulated in the seed’s embryo and used in the first stages of germination (Bailly 2019; Ciacka et al. 2020, 2022b). To date, the RNS action during seed deterioration has not been fully explored. The “nitrosative door” model presents the bimodal action of RNS in seeds (Krasuska and Gniazdowska 2012; Krasuska et al. 2015; Ciacka et al. 2022a). The final molecular effect (positive or negative) of RNS that react with proteins, fatty acids and nucleic acids depends on the concentration of these reactive compounds. The physiological state of the organism is also relevant in this consideration (Ciacka et al. 2022a). Data on the biological action of NO/RNS in the improvement of the quality of aged seeds are rather scarce. The reduction of the negative effect of accelerated ageing of elm (*Ulmus pumila* L.) seeds was achieved using NO donors application before ageing treatment (He et al. 2018). Similarly, the use of NO donors after the artificial ageing of oat (*Avena sativa* L.) seeds enhanced their vigour (Mao et al. 2018). Prolonged warm stratification at 25 °C (conditions consistent with accelerated ageing) of apple (*Malus domestica* Borkh.) seeds resulted in a decrease in their vigour associated with embryo deterioration and a reduction in NO emissions from the embryonic axes (Dębska et al. 2013). Short-term fumigation with NOx of apple embryos isolated from aged seeds improved their germination in comparison to the control (non-treated embryos) (Ciacka et al. 2022b). ROS, a part of a cellular signalling system, are divided into free radicals e.g. superoxide anion (O_2_^•−^), generally characterised by higher reactivity and instability, and non-radical molecules like hydrogen peroxide (H_2_O_2_) with lower reactivity and longer half-life (Mittler 2017; Considine and Foyer 2021). These compounds regulate the growth and development of plants (Considine and Foyer 2021). ROS influence the cell components directly, and/or are involved in oxidation–reduction (redox) reaction that enables sensing and adaptation to stress conditions. The maintenance of the cellular ROS balance is crucial for proper metabolism. Therefore, the content of each highly reactive molecule of physiological importance must be carefully controlled (the maintenance of homeostasis).

The conditions of low water content in the mature seeds are not conducive to enzymatic activity, so any repair mechanisms are related to the presence of non-enzymatic molecules, which are usually regulators of the redox potential. An important part of the “redox hub” — the core linking the cellular energy system and the net of signalling pathways (Foyer and Noctor 2011) are compounds with cysteinyl (Cys) residues. The biological activity of thiol groups (–SH) in the side chain of Cys depends on the chemical properties of sulphur atoms, which play a key role in reversible redox reactions together with redox signalling (Considine and Foyer 2021). Proteins containing Cys are prone to multiple posttranslational modifications (thiol-based oxidative PTMs) such as glutathionylation or *S*-nitrosation, which can affect the metabolism of various molecules such as glutathione or nicotinamide adenine dinucleotide phosphate (NADPH) (Corpas et al. 2022).

The reduced form of glutathione (GSH: γ-glutamyl cysteinyl glycine), the crucial element of a non-enzymatic, ROS-modulating system, is a water-soluble, low-molecular-weight thiol. As an electron donor, this evolutionary old molecule stabilises free radicals and thus participates in the preservation of a cellular redox state (Kranner et al. 2006; Noctor et al. 2012). Glutathione, located in the cytosol, nucleus, mitochondria and chloroplasts, is a vital compound for seeds (Cairns et al. 2006; Diaz-Vivancos et al. 2015), especially since orthodox seeds may lose ascorbic acid (another non-enzymatic antioxidant compound) during the desiccation phase, and the amount of which in dry seeds is minimal (Tommasi et al. 1999). GSH is the most abundant non-protein thiol in aerobic organisms, as its concentration can be measured even in mM (Noctor et al. 2012), which indicates its unique value. The oxidised form of GSH is GSSG. By controlling ROS content, GSH acts as a regulatory molecule responsible for cell fate (Diaz-Vivancos et al. 2015). The standard redox potential of glutathione in pH 7 is − 240 mV (Noctor et al. 2012). Thus, the half-cell reduction potential (E_GSSG/2GSH_) may serve as a marker of the current physiological state of the cells. Elevated values of this indicator point to the stress conditions or ageing progress. The values range between − 180 and − 160 mV is linked to the initiation of irreversible lethal alterations of the cell (Kranner et al. 2006).

ROS detoxification leads to GSH oxidation and GSSG formation. Such conversion of glutathione may be enzymatically stimulated via the activity of the heme-free thiol peroxidases — glutathione peroxidase (GPX; EC 1.11.1.9) (Pei et al. 2023). GPX utilises GSH and/or other reducing equivalents (Ursini et al. 1995). Depending on the amino-acid sequence, substrate specificity and the differences in the tissue expression, there are eight members of the mammalian GPX family. GPX1 location is the cytosol, GPX2 is the gastro-intestinal enzyme, GPX3 is presented in the plasma, GPX4 scavenges the phospholipid hydroperoxides, and seleno-independent GPX5 is located in the epididymis (Chu 1994; Drevet 2000). GPX6 is only expressed in the human body. The active site of GPX5 and GPX7-GPX8 lacks selenium Cys (SeCys) and is replaced by Cys (Pei et al. 2023). In plants, GPX-like enzymes are not SeCys proteins. There is no clear scientific evidence for the existence of a UGA codon (opal codon) in the gene sequences, responsible for the insertion of a SeCys (Herbette et al. 2002 and citations therein). However, the occurrence of the selenium-dependent GPX in plants cannot be excluded (Fu et al. 2002). The presence of Cys in plants’ GPX-like active site lowers its peroxidase activity compared to the animal SeCys isoforms (Herbette et al. 2002 and citations therein). Nevertheless, GPXs-like were localised in various cellular compartments of Arabidopsis (cytosol, mitochondria, chloroplasts, peroxisomes, and apoplast) (Rodriguez Milla et al. 2003), which may prove their special role in plant cells. In plants, alterations in the GPX-like activity as a reaction to the stress factors have been shown (Fu et al. 2002). Moreover, higher activity of this enzyme during seed dormancy alleviation and germination was demonstrated (Krasuska and Gniazdowska 2012; Ciacka et al. 2022a).

The enzyme responsible for the recycling of GSSG to GSH and the maintenance of the high level of GSH in cells is glutathione reductase (GR, EC 1.6.4.2). This enzyme is mostly located in the cellular compartments of high electron flux, i.e. plastids or mitochondria (Couto et al. 2016). GR is a flavoprotein oxidoreductase, which uses flavin adenine dinucleotide (FAD) and NADPH as cofactors that serve as an electron donor to reduce GSSG into GSH (Kranner et al. 2006; Noctor et al. 2012). Increased expression of GR is noted during stress conditions, as was observed for various plant species (Gill et al. 2013). Moreover, it was demonstrated, that apple seed dormancy breakage was accompanied by higher GR activity (Krasuska and Gniazdowska 2012; Ciacka et al. 2022a).

Apple seeds are characterised by deep dormancy, and as they belong to the *orthodox* type, they are subjected to intensive desiccation during seed maturation (Lewak 2011). Due to the low water content, the state of very low metabolic activity inhibits germination and maintains high seed vigour but does not entirely prevent ageing. Slowly progressing deterioration processes of *orthodox* seeds force the use of different laboratory protocols to speed up ageing. However, the effects of such procedures do not fully reflect the changes occurring in seeds during their long-term storage, e.g. in seed banks (Roach et al. 2018). The advantage of using ageing protocols is to fasten the appearance of metabolic alterations resulting in the accumulation of toxic compounds. This is somewhat of a compromise in the ageing research, especially useful for *orthodox* seeds. For apple seeds, the protocol of accelerated ageing was described in detail by Ciacka et al. (2022b).

As we demonstrated previously, apple embryos isolated from aged seeds, shortly treated with NOx, were characterised by a higher germination rate and a higher total free radicals removal capacity. NOx application implicated ROS metabolism, lowering the level of total oxidised RNA (Ciacka et al. 2022b). Furthermore, NOx fumigation of embryos of artificially aged apple seeds increased the level of phenylalanine and tyrosine, mitigating the accumulation of *meta*-tyrosine, a non-protein amino acid, whose level increased in a highly oxidative environment (Ciacka et al. 2022c). Such treated, aged embryos were also characterised by alterations in the expression of genes associated with the regulation of degenerative processes (Ciacka et al. 2022c, b). The results indicate that NO may be a molecule that improves the quality of aged seeds. This effect is most likely related to the modulatory role of NO on ROS metabolism.

NO as a “remedy” against seed ageing, regulates the detrimental effect of ROS accumulation. Taking into account that GSH prevents redox imbalance during oxidative stress, it is valuable to combine the role of NO as a molecule that mitigates the effects of seed ageing with glutathione metabolism. Thus, in the current work, we focussed on the influence of NOx on GSH and GSSG content, GSH:GSSG ratio and values of E_GSSG/2GSH_ in the axes of apple embryos isolated from seeds subjected to artificial ageing for 7, 14, and 21 days, cultured for 48 h under optimal conditions. We analysed transcript levels of genes encoding GPX-like and GR, together with the enzymatic activity. The aim of our work was to link the beneficial role of NO in the maintenance of seed vigour of aged *orthodox* seeds with a multifaceted compound — glutathione. Publications on the participation of the glutathione system in *orthodox* seeds, especially in terms of NO impact on seed ageing are unique.

## Materials and methods

### Plant material

**Fig. 1:**
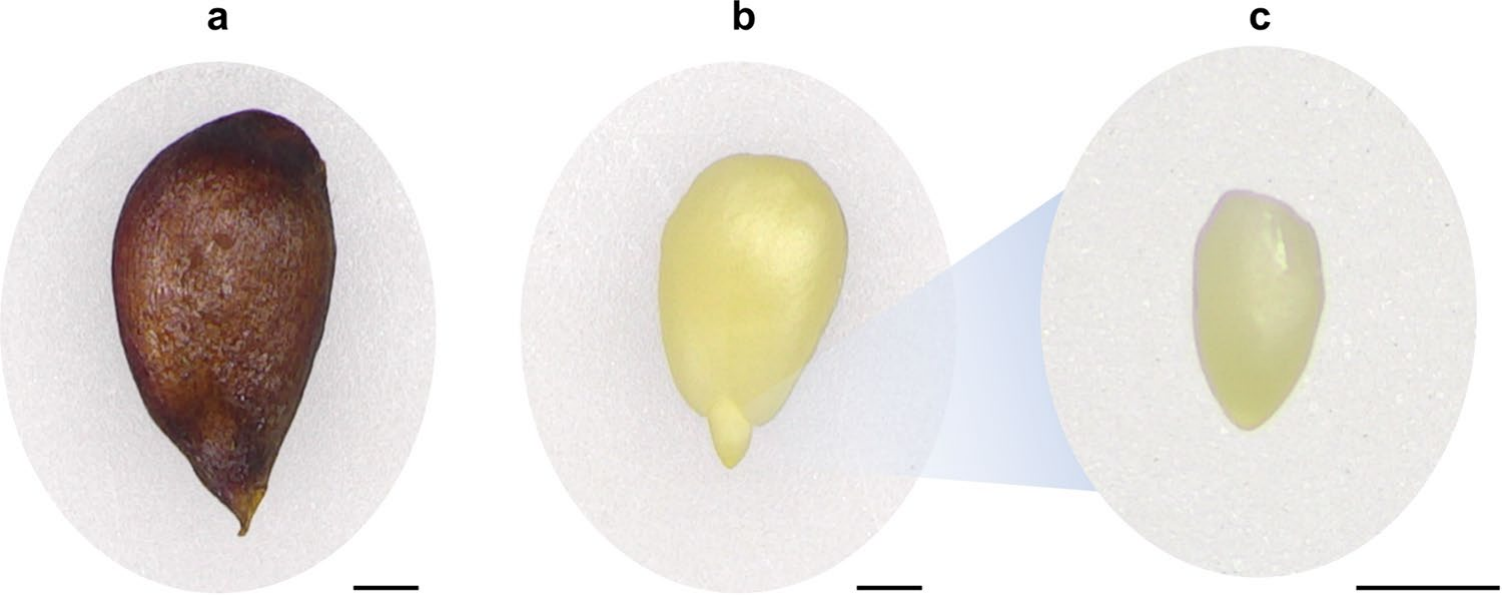
Representative photos of the experimental material. Apple seed (**a**), apple embryo (**b**), isolated apple embryonic axis (**c**). Bar = 1 mm

Experiments were conducted on axes of apple (*Malus domestica* Borkh. cv. Antonówka) embryos isolated from seeds (Fig. 1) subjected to accelerated ageing treatment, as described in Ciacka et al. (2022b). Fully ripe apple fruits were acquired from the same local orchard WIKPOL (Dąbrowa, Poland), harvested in 2022 and 2023. The seeds were manually isolated and dried out on air at room temperature. Before use, dried seeds were stored in a glass container at 5 °C for dormancy preservation. To assess seed quality, germination tests were performed each year on new seed material.

The procedure of accelerated ageing was carried out as follows: seeds were placed in glass Petri dishes (100 seeds per dish ⌀ 15 cm), mixed with sterile sand, and watered. The humidity was set to 50%. Seeds were stored at 35 °C for 7, 14 and 21 days. During the process, seeds were thoroughly stirred two times a week. After the ageing treatment, seeds were washed from sand, and seed coats were removed.

Isolated apple embryos were subjected to short (3 h) treatment with NOx. Embryos (50 per container) were placed in a sealed glass container (0.5 L) and fumigated with gaseous NOx produced in a reaction of 14.5 mM NaNO_2_ with 0.2 M HCl (Yamasaki 2000; Ciacka et al. 2022b). After the procedure, embryos were rinsed with distilled water. To assess whether a given seed ageing treatment produces similar effects, germination tests of control and NOx-treated embryos isolated from aged seeds were performed.

Embryos treated with NOx and those non-treated (the control, embryos of aged seeds) were placed in Petri dishes filled with moistened filter paper for 48 h in a growth chamber (Sanyo MLR-35OH) at 25/20 °C day/night, a 12/12 h photoperiod, the light intensity of 100 µmol PAR m^−2^ s^−1^. After 48 h, the embryonic axes were isolated from the embryos and used in the experiments.

### Germination test

Apple embryos isolated from aged seeds, non-treated (the control) and NOx-treated (NO) were placed in Petri dishes (15 embryos per dish ⌀ 9 cm) filled with moistened filter paper with water. In addition, aged embryos were placed in Petri dishes on filter paper moistened with 1 mM GSH water solution. Embryo culture was performed in a growth chamber (Sanyo MLR-35OH, in conditions described above) for 7 days. Every day, observations of germinating embryos were made. The number of germinated embryos (with gravitropic bending of the embryonic root), the uniformity of seedling cotyledons’ greening, and the seedlings’ general phenotype were noted. The 1 mM solution of GSH was changed every 2 days. Germination tests were repeated three times.

### Measurement of GSH and GSSG content

For the measurement of GSH and GSSG concentration, the method of separation with the reversed-phase HPLC and fluorescence detector was used. The procedure was conducted according to Kranner (1998) with modifications.

Approximately, 30–35 embryonic axes (30 mg) were grounded in liquid nitrogen. The obtained tissue powder was transferred to the Eppendorf probes and homogenised with 100 mM HCl, 2% (w/v) PVPP and 1 mM EDTA. Then, the samples were vigorously vortexed, incubated at RT for 20 min and centrifuged (4 °C, 12,000 *g*, 20 min).

Determination of total glutathione content was conducted by mixing 20 μl of obtained supernatant with 30 μl of 200 mM CHES (Sigma-Aldrich, C8210) buffer (pH 9.4) and 5 μl of 3 mM DTT (Roth, 6908.1), followed by 1-h incubation at RT. For derivatisation, 3.4 μl of 15 mM monobromobimane (MBBr; Sigma, 69,898) was added. The reaction was carried on for 15 min at RT in darkness until it was terminated by the addition of 3.4 μl of 20% acetic acid. Into the HPLC system, 10 μl of the sample was injected.

Measurement of GSSG concentration was performed by mixing 133 μl of the obtained sample with 10 μl of 50 mM N-ethylmaleimide (NEM, Acros organics, 2928) and 200 μl of 200 mM CHES. To discard the NEM residues, the mixture was combined with 350 μl of toluene (Sigma-Aldrich, 203-625-9) and properly vortexed for 30 s. After the separation of phases, the toluene phase was disposed of. Removal of excess NEM was performed 6 times. Then, 50 μl of the sample was mixed with 5 μl of 3 mM DTT and incubated for 1 h at RT in darkness. Derivatisation of samples was performed as described above.

Separation of bromine derivatives was performed with Bionacom Velocity C18 LPH (4.6 ˟ 150; 3 μm) column held in the oven set at 35 °C. For peak detection, the FP-2020/2025 Intelligent Fluorescence Detector (JASCO) (Ex 390 nm; Em 478 nm) was used. As mobile phases, 0.25% acetic acid containing 5% methanol, adjusted to pH 3.9 with 5 M NaOH (A) and 100% methanol (B) were used.

The gradient program used for sample separation: 0–5 min 80% A, 5–30 min 80–75% A, 30–38 min 75–70% A, 38–45 min 70–0% A, and 45–50 min 0–80% A. The flow of the mobile phase was set at 1 ml min^−1^.

The standard curve was prepared with GSH as a standard and processed as described above. Measurements were done in four biological replicates, each in two technical repetitions. Total glutathione pool, GSH and GSSG content were presented as nmol g^−1^ FW.

### Glutathione half-cell reduction potential

E_GSSG/2GSH_ (in mV) was calculated based on the Nernst equation (Kranner et al. 2006; Morscher et al. 2015) as described in Ciacka et al. (2019):

$$E_{GSSG/2GSH} = - E^{0{\prime} } - RT\,[nF]^{ - 1} 1n\,[GSH^2 GSSG^{ - 1} ]$$

where R is the gas constant (8.314 J K^−1^ mol^−1^); T is the temperature in K during ageing (35 °C); n is the number of transferred electrons (2GSH → GSSG + 2H^+^ + 2e^−^); F is the Faraday constant 9.6485 10^4^ C mol^−1^; E0′ is the standard half-cell reduction potential of glutathione (− 0.240 V). As for glutathione measurement, the calculations were done in four biological replicates, each in two technical repetitions.

### Measurement of GPX-like activity

The determination of GPX-like activity was performed according to Flohe and Günzler (1984) with modifications described in Krasuska and Gniazdowska (2012). Apple embryonic axes (25 mg) were homogenised in 50 mM K-phosphate buffer (pH 7.5), with 0.1% triton X-100, 5 mM DTT (Sigma, D0632), 5% (w/v) glycerol, 0.1% protease inhibitor cocktail (Sigma, P9599) and 2% (w/v) PVPP on ice. The sample was centrifuged at 13,000 g, 4 °C for 10 min and the supernatant was desalted using concentrator PES, 3 K MWCO (Thermo Scientific) at 13,000 *g*, 4 °C for 20 min. The obtained desalted supernatant (25 µl) was incubated with 125 µl of 50 mM K-phosphate buffer (pH 7.5), 25 µl 0.1 M aminotriazole, 25 µl of 2.5 mM GSH and 2.5 U of GR (Sigma G3664) for 10 min at 30 °C. Then, to the mixture, 12.5 µl of 3% H_2_O_2_ and 25 µl 2 mM β-NADPH (Sigma-Aldrich, N5130) were added.

The activity of GPX-like was measured as the decline in the absorbance at 340 nm. The measurement was performed with the microplate reader (Clariostar Plus, BMG LabTech). The GPX-like activity was expressed as nmol NADPH min^−1^ mg^−1^ protein. Experiments were done in three biological replicates, in three technical repetitions.

### Measurement of GR activity

The activity of GR was determined as described by Esterbauer and Grill (1978) with modifications. Apple embryonic axes (25 mg were homogenised in 50 mM K-phosphate buffer pH 7.5 containing 5 mM DTT, 5% (w/v) glycerol, 0.1% (w/v) triton X-100, 0,1% (v/v) protease inhibitor cocktail and 2% (w/v) PVPP on ice. Then, the sample was vortexed, centrifuged (13,000 g, 4 °C for 10 min) and the supernatant was desalted with centrifugal concentrator PES, 3 K MWCO (Thermo Scientific) at 13,000 *g*, 4 °C for 20 min.

Protein extract (40 μl) was added to 175 μl of K-phosphate buffer pH 7.5 and 25 μl of 5 mM GSSG (Sigma G-6654). Then, the mixture was incubated at 30 °C for 10 min. The reaction was induced by 25 μl of 2 mM NADPH and was carried out at 30 °C for 5 min. The activity of GR was measured as the decline in absorbance at 340 nm (microplate reader Clariostar Plus, BMG LabTech). Simultaneously, the reaction was held without GSSG addition. GR activity was expressed as nmol NADPH min^−1^ mg^−1^ protein. Experiments were conducted in three biological replicates, in three technical repetitions.

### Measurement of protein concentration

For calculations of enzymatic activity, the protein concentration in the apple embryonic axes was determined using a Bradford reagent (Bradford 1976). The standard curve was prepared with serum bovine albumin (BSA — Sigma, A7030) as a standard.

### Gene expression analysis

The measurement of gene expression in the axes of apple embryos was done using Bio-Rad CFX Connect™ Real-Time PCR Detection System Total RNA was isolated using RNAzol® RT (Sigma, R4533), according to the manufacturer’s guidelines. 200 ng of total RNA was taken for cDNA synthesis. The cDNA was synthesised using the RevertAid First Strand cDNA Synthesis Kit (Thermo Scientific, #K1622) in a total volume of 10 μl. The final product was diluted 3.5 times. iTaq™ universal SYBR® Green supermix (Bio-Rad, #172–5124) was used for the reaction in a total volume of 12 μl (6 μl PCR supermix, 1 μl cDNA, 1 μl primer, 4 μl H_2_O). Specific primers were designed based on nucleotide sequences available in the GenBank database on the National Center of Biotechnological Information (NCBI) and in the Genome Database for Rosaceae (https://www.rosaceae.org/) (Table S1). Gene names were defined based on similarity to the sequences of Arabidopsis genes.

Expression levels were normalised using two reference genes and calculated using the method described by Vandesompele et al. (2002) and Hellemans et al. (2007). Experiments were done in three biological replicates, in three technical repetitions.

### Statistics

Data were analysed using the Statistica13 Software. All data were obtained in at least 3 independent experiments with at least 2 repetitions each and presented as mean values ± SD. After two-way ANOVA, homogenous groups were evaluated using the Duncan’s test. For the gene expression analysis, the *t* test was used for group comparison.

## Results

### Ageing effect of apple seeds was mitigated by the treatment with NOx or GSH

**Fig. 2:**
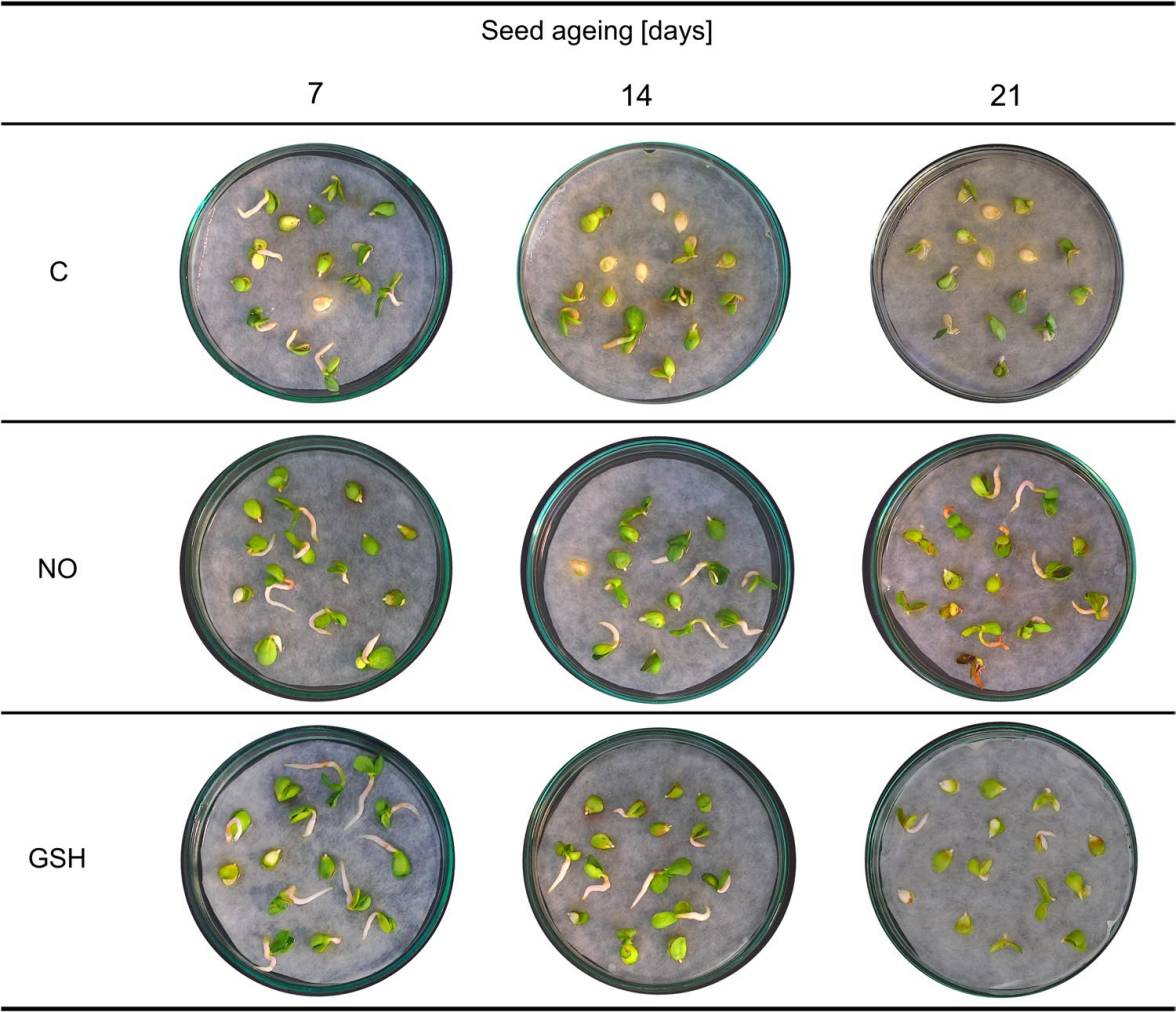
Representative photos of apple seedlings developing from germinating embryos isolated from seeds subjected to accelerated ageing for 7, 14 and 21 days, after 7 days of the culture under optimal conditions (detailed description in the text). Control embryos (C), embryos briefly treated with NOx (NO) and embryos germinating in the presence of 1 mM GSH (GSH). Germinating tests were conducted three times

Germination test of embryos isolated from aged seeds revealed numerous alterations in embryo/seedling morphology (Fig. 2). The process of ageing resulted in deterioration of seed vigour, which was manifested by rotten embryos, or non-germinated (with inhibited axes’ elongation) embryos with green cotyledons, or seedlings with only one green cotyledon. The detrimental effect of ageing was intensified as the procedure was prolonged (14 and 21 days). NOx fumigation led to an improvement in the vitality of developing seedlings. Although not reversed, the ageing effect was mitigated by NOx treatment, especially after 14 and 21 days of ageing. Seedlings developed from embryos subjected to GSH water concentration, similarly to those treated with NOx exhibited less morphological abnormalities and did not manifest such tissue degeneration as the control (Fig. 2).

### NOx and GSH enhanced the germination of embryos isolated from aged apple seeds

**Fig. 3:**
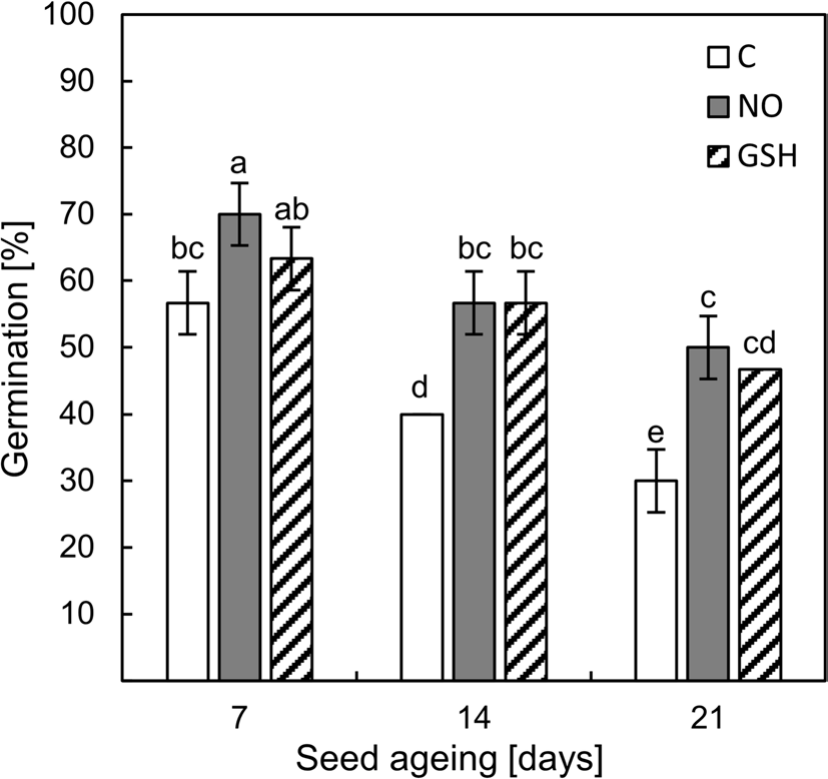
Germination of apple embryos isolated from seeds subjected to accelerated ageing for 7, 14 and 21 days, after 7 days of the culture under optimal conditions (detailed description in the text). Control embryos (C), embryos briefly treated with NOx (NO) and embryos germinating in the presence of 1 mM GSH (GSH). Values are an average of three biological repetitions ± SD. The homogeneous groups were labelled with lowercase letters, according to the Duncan’s post-hoc test (*P* < 0.05)

Apple embryos isolated from seeds subjected to 7-day accelerated ageing after 7 days of the culture germinated in approx. 60% (Fig. 3). NOx treatment significantly increased the germination of these embryos, up to 70%. Using a GSH solution, only a slight and insignificant increase in germination was observed (65%).

Extending the duration of ageing (14 and 21 days — control) led to a reduction in the number of germinated embryos to 40% and 30%, respectively (Fig. 3). Fumigation with NOx as well as GSH application improved the germination of embryos isolated from seeds aged for 14 days by 40%. After 21 days of ageing, both embryo treatments resulted in an increase in germination by 60%.

### NOx affected the level of glutathione after the shortest and the longest duration of seed ageing

**Fig. 4:**
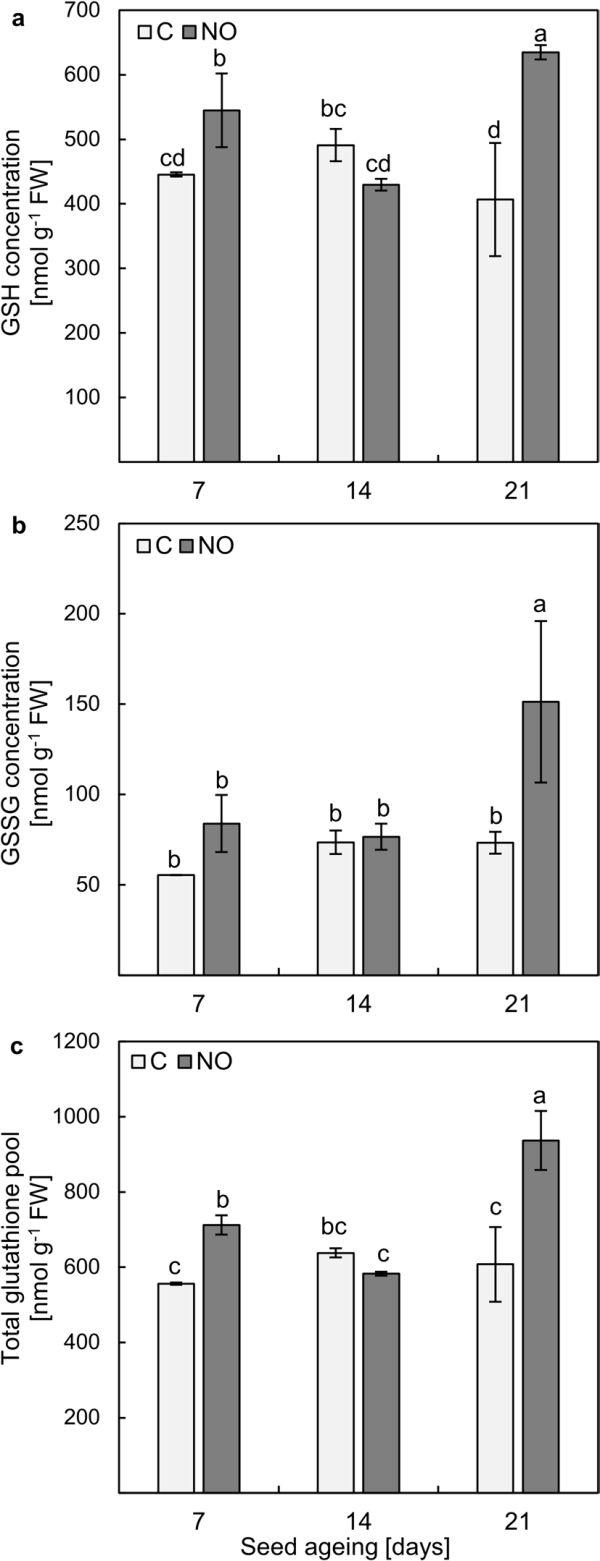
The concentration of GSH (**a**), GSSG (**b**) and total glutathione pool (**c**) in the axes of apple embryos isolated from seeds subjected to ageing for 7, 14 and 21 days (C—control) or the embryos isolated from aged seeds and treated with NOx. Values are an average of four biological repetitions ± SD. The homogeneous groups were labelled with lowercase letters, according to the Duncan’s post hoc test (*P* < 0.05)

The concentration of GSH in the axes isolated from embryos of apple seeds subjected to ageing for 7 days, after 48 h of embryo culture, was approximately 445 nmol g^−1^ FW and did not change significantly during prolonged ageing (Fig. 4a). Treatment of the embryos with NOx resulted in an increase in GSH content in the embryonic axes isolated from seeds aged for 7 and 21 days by 22% and 56%, respectively. No significant changes in GSH concentration were observed in the axes of NOx-fumigated embryos isolated from seeds aged for 14 days in comparison to the control.

In the embryonic axes, the level of GSSG did not differ regardless of how long the seeds were aged (Fig. 4b). Treatment of the embryos with NOx led to a slight increase in GSSG concentration after 7 days of ageing, but the result was not statistically significant. NOx stimulated the accumulation of GSSG only in the axes of embryos of seeds aged for the longest time. In this tissue, GSSG concentration increased 2 times in comparison to the control.

The concentration of the glutathione (both reduced and oxidised—the total glutathione pool) was similar regardless of the duration of seed ageing (Fig. 4c). NOx treatment changed the level of the total glutathione pool after the shortest and longest time of seed ageing. The level of total glutathione in the axes of NOx-treated embryos of seeds aged for 7 days increased by approximately 30% compared to the control. The most pronounced effect of NOx treatment was observed in the embryonic axes of seeds after 21 days of ageing, resulting in a 54% change.

### The GSH:GSSG ratio and EGSSG_/2GSH_ did not significantly differ after fumigation of aged apple embryos with NOx

Despite the slight changes in the average values of GSH:GSSG ratio and E_GSSG/2GSH_ in the axes of embryos of aged seeds, treated with NOx or not, statistical analysis did not reveal significant differences (Table 1).

**Table 1:**
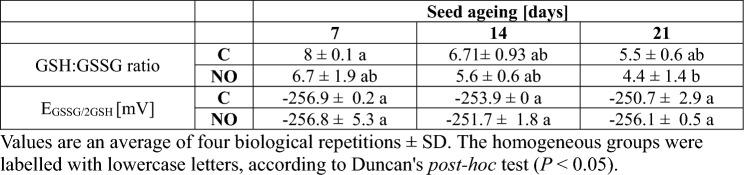
The GSH:GSSG ratio and half-cell reduction potential in the axes of apple embryos isolated from seeds subjected to ageing for 7, 14 and 21 days (C—control) or the embryos isolated from aged seeds and treated with NOx

### NOx improved GR and GPX activity in the embryonic axes of seeds subjected to accelerated ageing

**Fig. 5:**
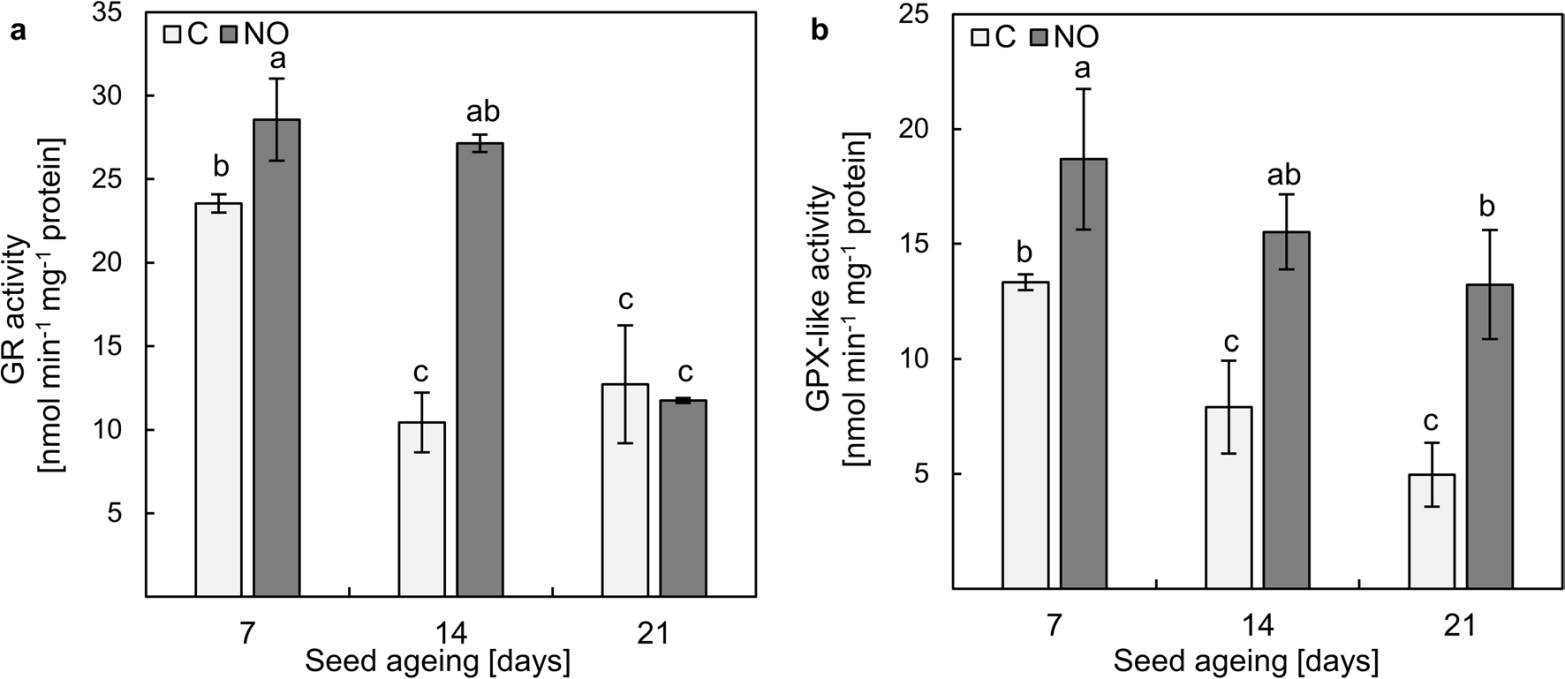
The activity of GR (**a**) and GPX-like (**b**) in the axes of apple embryos isolated from seeds subjected to ageing for 7, 14 and 21 days (C—control) or the embryos isolated from aged seeds and treated with NOx. Values are an average of three biological repetitions ± SD. The homogeneous groups were labelled with lowercase letters, according to the Duncan’s post hoc test (*P* < 0.05)

The accelerated ageing resulted in a decrease in GR activity in the embryonic axes (Fig. 5a). In the axes of seeds aged for 7 days, the activity was 23 nmol min^−1^ mg^−1^ protein. After 14 days of ageing, it decreased by more than twice and remained at the same low level after 21 days of the procedure. NOx treatment increased GR activity in the axes of seeds aged for 7 and 14 days. The enzymatic activity was higher by 21% and 160%, respectively. The stimulatory effect of NOx application on GR activity was not observed in the axes of seeds aged for 21 days (Fig. 5a).

In the embryonic axes, the activity of GPX-like decreased as a result of prolongation of the duration of ageing (Fig. 5b). After 7 days of ageing, the enzyme activity was 13 nmol min^−1^ mg^−1^ protein and it decreased by almost 41% after 14 days of ageing. After the longest ageing time, GPX-like activity did not differ from that in the tissue after 14 days of ageing. NOx treatment increased GPX-like activity regardless of the duration of seed ageing (Fig. 5b). In comparison to the controls, after 7 days of ageing, the enzyme activity was higher by 25%, after 14 days of ageing it was doubled, and after 21 days of seed ageing increased almost 3 times.

### NOx upregulated the expression of *GR* and *GPX* genes in the embryonic axes of aged apple seeds

**Fig. 6:**
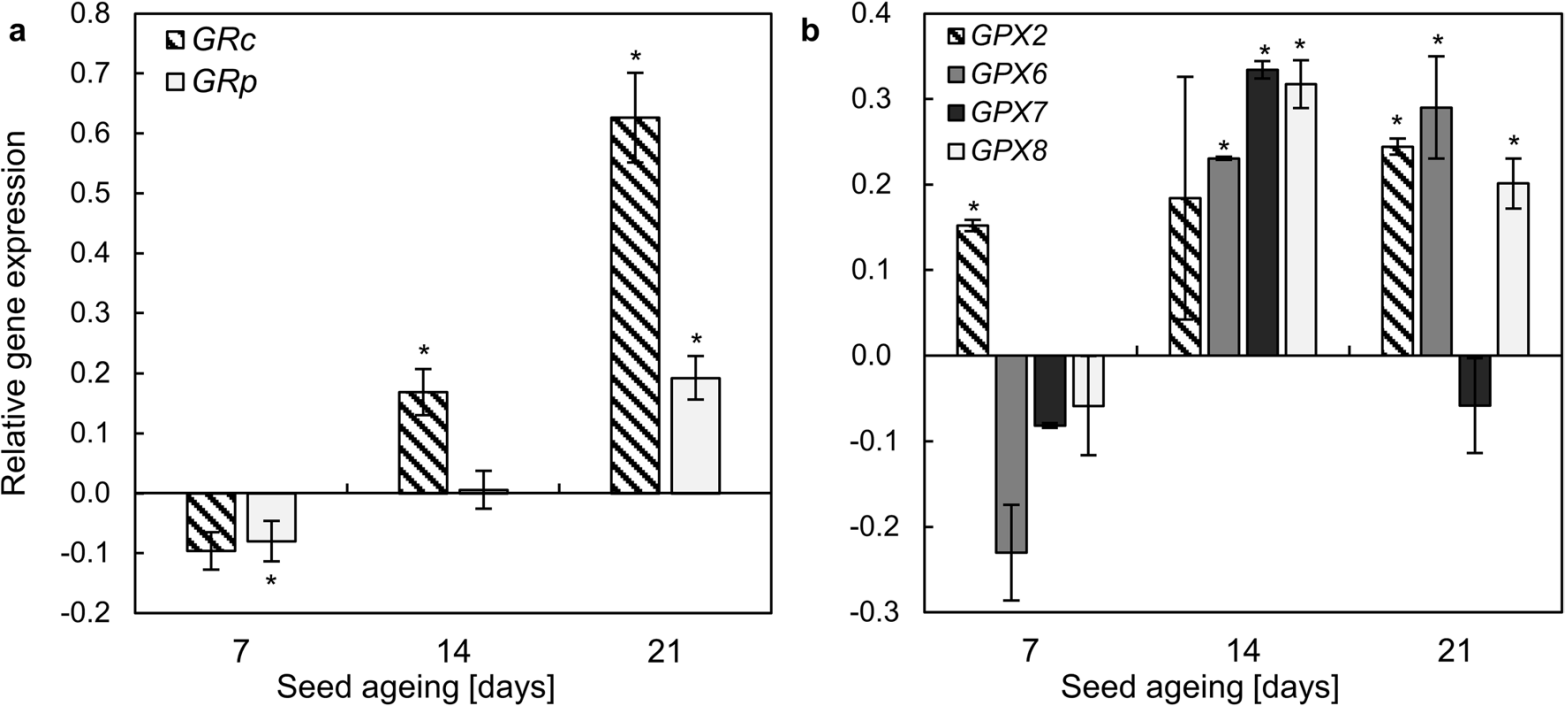
Changes in the expression of genes encoding GR homologues: *GRc* and *GRp* (**a**) and GPX: *GPX2*, *GPX6*, *GPX7*, *GPX8* (**b**) induced by NOx in the axes of apple embryos isolated from seeds subjected to ageing for 7, 14 and 21 days. Values are an average of three biological repetitions ± SD. Asterisks indicate significant differences between the treated sample compared to the control sample, obtained by the Student’s *t *test (*P* < 0.05)

The analysis of *GR* transcript levels concerned genes encoding the enzyme localised in the cytoplasm (*GRc*) and plastids (*GRp*) (Fig. 6a). Treatment of 7-day-aged embryos with NOx resulted in the downregulation of both analysed genes. After 14 days of ageing, NOx did not affect the transcript level of *GRp*; however, the level of *GRc* was upregulated by 0.17 units in comparison to the control. In the axes of seeds aged for 21 days, NOx increased *GRc* and *GRp* transcript levels by 0.6 and 0.2 units, respectively.

The transcript levels of genes encoding GPX-like (*GPX2*, *GPX6*, *GPX7*, *GPX8*) were analysed (Fig. 6b). Fumigation of 7-day-aged embryos with NOx resulted in the upregulation of only *GPX2.* The changes in transcript levels of other analysed genes were not significant. In the embryonic axes of seeds subjected to ageing for 14 days, NOx increased the expression of *GPX6*, *GPX7* and *GPX8*. After 21 days of ageing, as a result of NOx exposition, the upregulation of *GPX2*, *GPX6*, and *GPX8* was observed.

## Discussion

The rate of ageing of the *orthodox-*type seeds depends on the plant species and storage or environmental conditions. Factors conducive to dormancy preservation, like very slow metabolism resulting from low water content in tissues, allow such seeds to sustain vigour at a high level for a long time. Various molecular mechanisms associated with deep dormancy maintenance or induction of germination of apple seeds have already been well described in the literature (Lewak 2011; Dębska et al. 2013; Ciacka et al. 2019, 2022a). Thus, this experimental material was used in the investigation of the biology of the typical *orthodox* seeds in the context of seed ageing. We established a laboratory protocol for accelerated ageing. The effectiveness of the procedure was confirmed by the viability test (TTC test) and the germination test of isolated embryos (Ciacka et al. 2022b). Prolonged accelerated ageing (21 days) of apple seeds resulted in considerable viability loss and even death of almost 90% of isolated embryos after 40 days of seed ageing. We also confirmed the beneficial effect of short-term treatment with NOx of apple embryos isolated from aged seeds, which stimulated total antioxidant activity in the embryonic axes (Ciacka et al. 2022b). RNS-dependent mitigation of the negative outcomes of seed ageing persisted until the 21st day of accelerated ageing and was reflected in a higher germination rate of embryos and undisturbed growth of seedlings, compared to the aged and non-treated control. The results of our previous experiments indicate that the effect of exogenous NOx on apple embryo vigour improvement depends on the seed ageing duration. The same applies to the results presented in this manuscript, as shown in Figs. 2 and 3.

We analysed the impact of NOx application after 48 h of the treatment, while the embryos were cultured on distilled water, under optimal conditions for germination. Based on the obtained results, we concluded that this point of time of the experiment is optimal for observation of the symptoms of disturbance in embryo metabolism as a consequence of seed ageing. In parallel, the RNS-dependent recovery effect and NO-related metabolic alterations in the tissue subjected to ageing were also noticed at this measurement point. As was mentioned in the Introduction section, RNS and other reactive NO derivatives are characterised by a high capacity to alter the structure and function of various functional molecules of cells. These changes include, among others, nitration (tyrosine or tryptophan residues) or *S*-nitrosation, mainly of proteins or peptides with -SH groups (Möller et al. 2019; Ciacka et al. 2022b). Besides the biochemical importance of ONOO^−^ in living systems, newly confirmed in plant cells, HNO react with thiol-containing proteins/peptides (Arasimowicz-Jelonek et al. 2022). Moreover, this molecule is involved in the regulation of the ageing process, as was demonstrated for Arabidopsis nitroxyl-pre-treated leaves, characterised by lower accumulation of senescence-associated gene transcripts. The kinetics of HNO depend on the redox status of the cell, and reductive conditions promote the formation of this NO derivative (Arasimowicz-Jelonek et al. 2022).

In this work, we focussed on the exogenous RNS impact on glutathione (GSH and GSSG) pool and metabolism in the axes of aged apple embryos, which is responsible for maintaining cell viability, redox signalling and various biochemical processes. GSH is engaged in the regulation of the thiol-disulphide status of the proteins that affect their function. This may especially concern redox-sensitive Cys residues in proteins and peptides, which have low pKa values (3–6) and are known as acidic thiols. Such thiols are highly susceptible to ROS and/or RNS attacks (Diaz-Vivancos et al. 2015). Data on the use of GSH to improve the quality of aged seeds are limited. The positive impact of the application of GSH on the germination of aged *Elymus sibiricus* (L.) seeds was noted (Yan et al. 2017). In addition, artificially aged oat seed primed with 1 mM GSH was characterised by a higher germination rate in comparison to the non-primed control (Xia et al. 2020). In our study, we compared the effect of 1 mM GSH application on the germination of embryos isolated from aged seeds to the germination of the NOx-treated embryos isolated from aged apple seeds. The most beneficial effect was noted for embryos isolated from seeds subjected to artificial ageing for 14 and 21 days. Both the percentage of germinated embryos (Fig. 3) and typical seedling growth (Fig. 2) in the presence of 1 mM GSH or after NOx treatment were similar. The seedlings were characterised by the cotyledons’ greening and the embryonic root’s growth. Therefore, this result allows us to assume that the effects of seed ageing may be mitigated by the overlapping actions of both NOx and GSH.

**Fig. 7:**
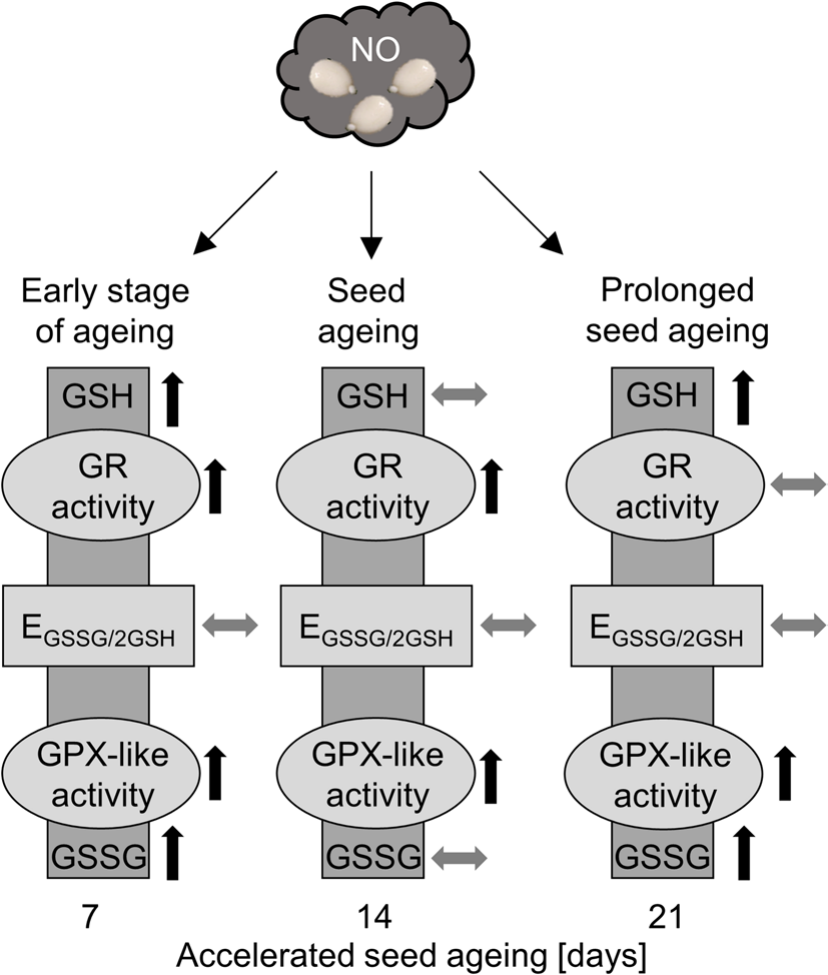
The role of NO and NO derivatives in embryo vigour maintenance by alteration of glutathione metabolism in the axes of embryos of aged apple seeds. The ageing process was divided into three phases. The state of an embryo isolated from 7-day-aged seeds is considered an early stage of ageing; after 14 days of accelerated ageing, symptoms of the deterioration processes are observed; prolonged seed ageing refers to advanced detrimental events

The oxidation of GSH during seed ageing is recognised. The alterations in GSH and GSSG levels were described for tomato (*Solanum lycopersicum* L.) seeds undergoing long-term storage or artificial ageing (De Vos et al. 1994). Artificial ageing of tomato seeds was accompanied by a marked loss of GSH and an increase in GSSG content. The decline in GSH concentration was observed for barley (*Hordeum vulgare* L.) seeds undergoing ageing under seed bank conditions and controlled deterioration treatment (CDT) — the type of artificial ageing protocol (Roach et al. 2018). The decrease in GSH and the increase in GSSG were also noted for aged pea (*Pisum sativum* L.) seeds (Kranner et al. 2006). The extension of the duration of CDT (21 days) of sunflower (*Helianthus annuus* L.) embryos (dormant and non-dormant), regardless of the O_2_ concentration in the environment, was accompanied by the decrease in total glutathione content, with a simultaneous increase in GSSG level in the embryonic axes (Morscher et al. 2015). In the case of apple embryos isolated from seeds artificially aged for 7, 14 and 21 days, after 48 h of the embryos culture, no particularly significant changes in the GSH content (Fig. 4a), and only a slight increase in GSSG level after 14 and 21 days of seed ageing (Fig. 4b) were observed. It has been noted that the higher GSSG content occurs in aged seeds of different plant species before their imbibition as a consequence of increased ROS levels and intensified oxidation reactions (Kranner et al. 2006; Morscher et al. 2015; He et al. 2018). During the imbibition of aged and non-aged tomato seeds, a serious decrease in GSSG content was demonstrated. In parallel, the significant increase in GSH content in non-aged tomato seeds, after 3 days of imbibition, was linked to the visible symptoms of germination (De Vos et al. 1994). Although short-term NOx treatment of the embryos isolated from 14-day-aged apple seeds did not alter GSH and GSSG levels in comparison to the control (Fig. 4a, b), it increased the GSH level (Fig. 4a) as well as the total glutathione pool (Fig. 4c) after the shortest and the longest time of ageing. The increase in the GSH content in such tissue may point to the induction of the germination process. Especially that, the enhanced GSH level was reached by fully non-dormant, ready-to-germinate apple embryos (Ciacka et al. 2022a). Moreover, short-term (3 h) treatment of dormant non-aged apple embryos with NOx resulted in stimulation of their germination (Gniazdowska et al. 2010). The slight decrease in GSH content in the axes of NOx-fumigated apple embryos isolated from seeds aged for 14 days confirms our observations regarding the variability of RNS action depending on the duration of the seed ageing treatment (Fig. 7) (Ciacka et al. 2022b). One probable explanation is the possibility of *S*-nitrosoglutathione (GSNO) formation, which acts as an intracellular NO reservoir and transporter (Corpas et al. 2013). The change in GSNO content was analysed in the axes of apple embryos during the transition from the dormant to the non-dormant stage, revealing an increased level of this compound characteristic of embryos capable of germination (Ciacka et al. 2019). In elm seeds, the use of NO donors before applying the ageing protocol increased the level of the *S*-nitrosated group (He et al. 2018). In desiccated *recalcitrant Antiaris toxicaria* (Pars.) Lesch. seeds that were NO fumigated, intensified *S*-nitrosation of antioxidant enzymes was noted (Bai et al. 2011). This effect was linked to higher germination rate. It can be assumed that *S*-nitrosation is a universal NO mechanism in improving seed quality. Seed ageing is connected with ROS accumulation; therefore, the measurement of GSNO concentration and the level of *S*-nitrosated proteins in the experimental model described in this work have to be considered in future studies.

The elevated glutathione pool (GSH + GSSG) (Fig. 4c) may indicate a higher antioxidant capacity and intensified total metabolic activity of the cells of the aged apple embryonic axes as a result of the RNS-dependent recovery effect. NOx treatment of elm seeds, before exposing seeds to accelerated ageing increased the GSH:GSSG ratio, compared to the aged (non-treated) control, and a decrease in value of the parameter was obtained when NO scavenger (c-PTIO) was used (He et al. 2018).

We also analysed E_GSSG/2GSH_ as a viability marker (Table 1). After 24 days of CDT of sunflower dormant and non-dormant embryos characterised by decreased germination capacity up to 50% and 60%, respectively, the E_GSSG/2GSH_ reached approx. − 150 mV (Morscher et al. 2015). Although the ageing of apple seeds was also associated with reduced germination capacity of isolated embryos and an increased number of rotten embryos (after 7 days of the culture), the embryos (both non-treated and NOx-treated) remained viable after 48 h of their culture (Ciacka et al. 2022b). Additionally, short-term NOx treatment of apple embryos isolated from aged seeds resulted in a higher percentage of germinated embryos and their higher viability (Ciacka et al. 2022b). The values of E_GSSG/2GSH_ in the axes of aged apple embryos non-treated and shortly treated with NOx remained constant and reached a low value of approx. − 250 mV (Table 1). Thus, the obtained results for E_GSSG/2GSH_ confirmed the results of the TTC test for such embryos (Ciacka et al. 2022b).

The stimulatory effect of NOx treatment on non-aged, dormant apple embryos on GR and GPX-like activity has been demonstrated, and an increase in activities of these enzymes was correlated with the progression of germination sensu* stricte* (Krasuska and Gniazdowska 2012). Accelerated ageing of apple seeds resulted in decreased GR activity (Fig. 5a). Short-term NOx treatment of apple embryos isolated from seeds aged for 7 and 14 days stimulated GR activity in the embryonic axes after 48 h of the embryos' imbibition. The increase in GR activity was also obtained in aged oat seeds as a result of sodium nitroprusside (NO donor) treatment (Mao et al. 2018). Despite higher levels of transcripts encoding GR in the axes of NOx-treated embryos isolated from apple seeds subjected to accelerated ageing for 21 days, the activity was comparable to the control (Fig. 5a). This result was accompanied by a relatively high level of GSSG in this tissue (Fig. 4b). During the artificial ageing of oat seeds, up to the 40th day of seed ageing, GR activity decreased and finally on the 42nd day of the ageing increased, reaching the level of GR activity in non-aged seeds. In parallel, GSH and GSSG contents decreased gradually, but for GSSG the downward trend was more considerable (Sun et al. 2022). This was reflected in the gradual increase in the GSH:GSSG ratio during the prolongation of seed artificial ageing (Sun et al. 2022). As GR depends on the availability of NADPH, we propose that after prolonged conditions of higher temperature and humidity, progressive metabolic changes may be related to the depletion of this nucleotide. However, regardless of the lower GR activity recorded, GSH content was elevated (Figs. 4a, 5a), which may suggest an enhanced GSH biosynthesis. After the ageing of NO pre-treated elm seeds, the upregulation of the expression of genes encoding enzymes of glutathione formation (γ-glutamylcysteine synthetase and glutathione synthetase) was noticed (He et al. 2018). Moreover, priming the aged oat seeds with 1 mM GSH stimulated GR activity (Xia et al. 2020), indicating that increased GSH levels may have a positive effect on various antioxidant pathways in aged tissues.

As in the case of GR activity, the artificial ageing of apple seeds was associated with a decrease in GPX-like activity in the isolated embryonic axes. Moreover, NOx treatment of apple embryos isolated from these seeds had a stimulatory effect on GPX-like activity (Fig. 5b). In the embryonic axes of sunflower non-dormant and dormant embryos subjected to the CDT, GR activity slightly decreased but only under high O_2_ conditions (Morscher et al. 2015). Such conditions also implicated GPX-like activity, which was lower, compared to the GPX-like activity measured in the axes of embryos subjected to CDT under ambient O_2_. The ageing of sunflower embryos under ambient O_2_ increased the activity of GPX-like in comparison to the non-aged tissue (Morscher et al. 2015).

The alterations in the enzymatic activity, especially in the case of seeds, should be considered together with changes in transcript levels encoding these enzymes. Accelerated ageing is linked to the lower expression of transcripts encoding various proteins of the “repair system” (Ciacka et al. 2022b). Mature pea seeds subjected to accelerated ageing were characterised by lower transcript abundance of *GRc* and *GRp* (Yao et al. 2012). The authors suggested that ageing triggered the degradation of seed-stored transcripts, and proposed that this process also induced inhibition of de novo mRNA synthesis for these genes (Yao et al. 2012). NOx treatment of apple embryos isolated from aged seeds altered the levels of GR transcripts (Fig. 6a). After 7 days of ageing (and 48 h of the culture), NOx fumigation lowered levels of *GRp* compared to the control (Fig. 6a). However, a significant increase in *GRp* and *GRc* transcripts was observed after 14 and 21 days of seed ageing. The same trend was noted for GPX-like genes (especially for *GPX6* and *GPX8*) (Fig. 6b). NOx treatment of elm seeds also led to the higher expression of *GPX*, which may be the explanation for the alterations in GSH content during ageing (He et al. 2018). We propose that NOx supplementation of embryos from the 14th day of seed accelerated ageing influences (directly or indirectly) the transcript levels and expressions of encoding enzymes, resulting in a balanced metabolism of GSH, and maintaining the adequate GSH:GSSG ratio.

GR as well as GPX-like, supported by glutathione, are the essence of the repair machinery during the progression of cellular deterioration. GPX-like enzymes are involved in the detoxification of lipid hydroperoxides and other reactive molecules, and, as non-heme-containing proteins are less sensitive to hydrogen cyanide (HCN) or RNS (Krasuska and Gniazdowska 2012). This is of great importance, especially in the case of apple seeds, in which cyanogenic compounds are metabolised (Krasuska et al. 2023). GPX-like is also considered an enzyme involved in H_2_O_2_ signal transduction (rather than in its scavenging); therefore, it acts as a regulator of the expression of ROS detoxification-related genes in response to different stress conditions (Bela et al. 2015). Together with GSH, GPX-like is engaged in the regulation of cellular redox homeostasis. Ageing of *orthodox* seeds disturbs redox balance; hence, RNS may be considered as compounds that preserve a more reductive environment of the cells of embryonic axes. Moreover, we have already proposed that the effect of NO depends on the duration of apple seed accelerated ageing (Ciacka et al. 2022b). Thus, we suggest that after 14 days of the ageing of apple seeds, NOx application stimulates GR and GPX-like activities and probably promotes the further conversion of GSH, which, however, requires verification. After 21 days of accelerated ageing, metabolic changes resulting from the progressive deterioration process, alter the NO modulatory effect (stimulation of GSH synthesis).

## Summary and perspectives

This research emphasises the importance of the cooperation between glutathione and antioxidant enzymes of glutathione metabolism in maintaining seed vigour. Despite the constant GSH, GSSG levels and GSH:GSSG ratio, and the relatively low value of E_GSSG/2GSH_ in embryonic axes isolated from seeds subjected to artificial ageing, embryos exhibit diminished vigour while developing seedlings possess degraded tissues. No changes in the value of the glutathione-related parameters are the manifestation of the decrease in GPX-like and GR activities, which may result in impairment of ROS homeostasis in aged tissue.

Moreover, the results confirm the earlier conjecture that the anti-ageing effects of RNS depend on the degree of tissue deterioration (Fig. 7). Up to the 14th day of ageing, NOx application stimulated the activity of GPX-like, accompanied by the simultaneous increase in GR activity, which prevents toxic GSSG accumulation. However, after extending the ageing protocol to 21 days, embryo fumigation with NOx provided potential support for ROS scavenging through increased GPX-like activity. Nevertheless, in this tissue, despite the upregulation of gene expression, no increase in GR activity (likely due to NADPH depletion) led to the accumulation of GSSG. Taking into account the previous studies, the beneficial effects of RNS against seed ageing have a time limit in the application of the treatment. Exceeding the limit synonymous with the advanced ageing processes, entailing irreversible cellular changes results in RNS no longer functioning as molecules exhibiting advantageous properties and even could promote tissue deterioration.

In addition, the conclusion we can draw is that the action of glutathione in the preservation of seed vigour should be considered comprehensively by analysing the entire glutathione metabolism. Considering that the findings from the research indicate additional potential applications for glutathione, an intriguing aspect for exploration would involve investigating the other pathways through which glutathione is engaged. This includes its potential role as a reservoir for NO or its involvement in reactions related to protein glutathionylation. This should be the subject of further investigation.

### Supplementary Information

Below is the link to the electronic supplementary material.

Supplementary file1 (PDF 131 KB)

## Data Availability

Data are available under reasonable request to the corresponding author.

## Abbreviations

GPX-like: Glutathione peroxidase-like
GR: Glutathione reductase
GSH: Reduced form of glutathione
GSSG: Oxidised form of glutathione
HNO: Nitroxyl
NOx: Nitric oxide derivatives
ONOO^−^: Peroxynitrite
RNS: Reactive nitrogen species
ROS: Reactive oxygen species
