# Supplementary material for: Reactive nitrogen species act as the enhancers of glutathione pool in embryonic axes of apple seeds subjected to accelerated ageing
Source: Planta. 2024 Jul 12;260(2):51. doi: 10.1007/s00425-024-04472-5 (PMC11245430; doi:10.1007/s00425-024-04472-5)
Supplement: Supplementary file 1 — Supplementary file1 (PDF 131 KB) [file 425_2024_4472_MOESM1_ESM.pdf]

Table S1 List of primers

Gene symbol	Primer sequence 5'-3'	Encoded protein	Gene ID (NCBI/ GDR)
Sar1	F: TTGATTTGGGCGGGCATCAGATTG<br>R: TCATCAGAGAGGAGAGCATCCAGC	small GTP-binding protein	MD04G1194800
Pdi	F: TGCTGTACACAGCCAACGAT<br>R: CATCTTTAGCGGCGTTATCCTTG	protein disulfide isomerase	XM_008344461.2
MdGRc	F:CTCTGTTATCATGTCGAGGAAGATGC<br>R:ATCTGGAGGCACGGACACCA	cytoplasmic glutathione reductase	XM_008382372.3
MdGRp	F: CCAAAGGTTGTGGCTCCTGA<br>R:ACATGCTGCATCTGTGCTCT	plastidic glutathione reductase	XM_008395622.2
MdGPX2	F:GTGGTTTAACACAATCCAACTACAAGG<br>R: GCAAGGAAACGCCAAAATCTCAAA	glutathione peroxidase	MD04G1034400
MdGPX6	F: TTCCCGGCAGATCCTTTTGTGGT<br>R: CTAGCCATTGTACGATTCAATCCG	glutathione peroxidase	XM_008394342.3
MdGPX7	F: GTTGAGAGATACCCACCAACGAC<br>R: TCAAGCTGCGACCAGTTTC	glutathione peroxidase	MD08G1055100<br>MD15G1042100
MdGPX8	F: TTTGGTGATGAGGAACCGGG<br>R: GGGACCAGCATTATCACCGT	glutathione peroxidase	XM_008385761.3
